# Synchronous multiple primary malignancies of clear cell renal cell carcinoma with sarcomatoid, thyroid carcinoma: a case report

**DOI:** 10.3389/fonc.2023.1174306

**Published:** 2023-06-27

**Authors:** Yaxian Tan, Xiaowen Chen, Mushi Ye, Xiaofang Li, Wenci Liu, Sihai Liao, Zhong Xie, Yufang Zuo

**Affiliations:** ^1^ Cancer Center, Affiliated Hospital of Guangdong Medical University, Zhanjiang, Guangdong, China; ^2^ Department of Urology, Affiliated Hospital of Guangdong Medical University, Zhanjiang, Guangdong, China; ^3^ Department of Pathology, Affiliated Hospital of Guangdong Medical University, Zhanjiang, Guangdong, China; ^4^ Department of Radiological Imaging, Affiliated Hospital of Guangdong Medical University, Zhanjiang, Guangdong, China

**Keywords:** papillary thyroid carcinoma, multiple primary malignancies, multidisciplinary, case report, clear cell renal cell carcinoma with sarcomatoid

## Abstract

Multiple primary malignant neoplasms (MPMNs) are defined as the presence of two or more malignancies with different histologies in the same patient. MPMNs are rare, accounting for fewer than 4% of all tumor cases. Depending on the time interval between the diagnosis of the different malignancies, they are classified as either simultaneous or metachronous MPMNs, with simultaneous being rarer in MPMNs. Here, we present a 63-year-old female patient presenting with multiple primary renal and thyroid carcinomas and discuss the risk factors, treatment options, and prognosis of rare dual carcinomas. We focus on managing multidisciplinary teams and selecting individualized treatment options to deliver valuable treatment strategies to patients.

## 1 Background

Multiple primary malignant neoplasms (MPMNs) are rare, with a reported incidence of 0.4% and 21% in the cancer population and a prevalence of 0.7% and 11% (1). Billroth initially proposed MPMN in 1889 (2). Subsequently, in 1932, Warren and Gates established the diagnostic criteria, which included the presence of two or more benign or malignant primary lesions exclusive of spread, recurrence, and metastatic disease (3). MPMNs whose interval between two primary cancers is within 6 months are called simultaneous MPMNs, whereas those with an interval of more than 6 months are called metachronous MPMNs. The incidence of double or triple carcinomas in patients with MPMNs is only 0.5% (1). The most common tumors in MPMN are found in the gastrointestinal tract, followed by the lung, head, and neck (4). However, dual primary malignancies of the kidney and thyroid gland are uncommon.

In total, this paper aims to report a case of synchronous clear cell renal cell carcinoma with sarcomatoid, and thyroid cancer, including the diagnosis and evaluation of which tumor to treat initially, to deepen the understanding of the disease and improve treatment outcomes.

## 2 Case presentation

### 2.1 Case report

A 63-year-old female patient presented to the Department of Urology for a left renal mass. Magnetic resonance imaging of the abdomen showed a 9.6-cm round-like soft tissue mass in the lower and middle parts of the left kidney, with the adjacent renal pelvis and calyces deformed by compression, a clear perirenal fatty space, and slightly enlarged retroperitoneal lymph nodes (**Supplementary Chart**). Hemoglobin (Hgb) was 49 g/L. We considered kidney cancer combined with bleeding. In combination with preoperative imaging, the patient was staged as cT2aN1M0, stage III, according to the 8th AJCC guideline. Previous tumor history, significant comorbidities, genetic risk factors, environmental exposures, and family history of cancer were all negative for the disease.

### 2.2 Diagnostic basis

**Figure 1:**
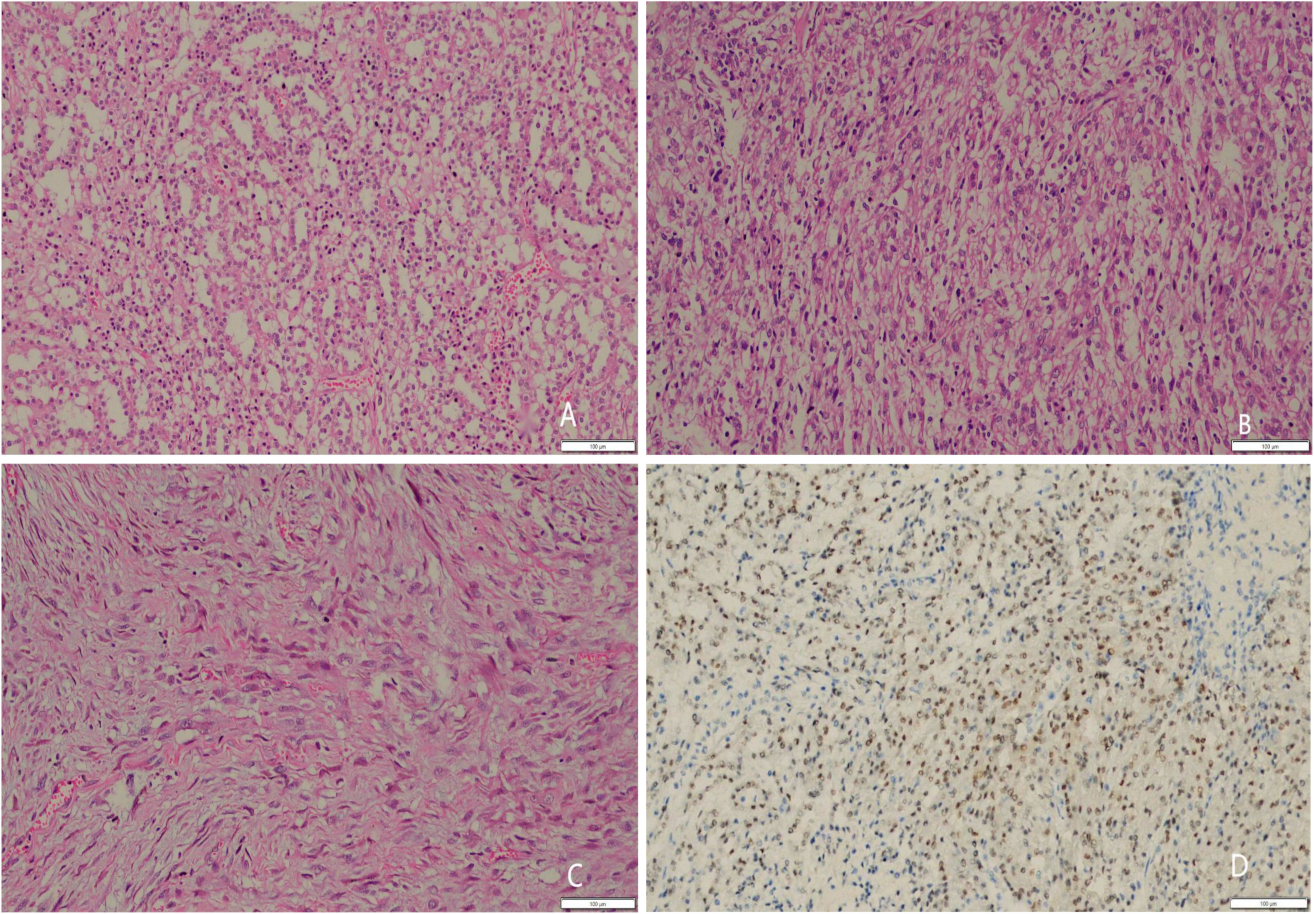
Under 200x microscope **(A)** Tubular area of renal carcinoma: tumor cells arranged in tubular shape, translucent cytoplasm, round nuclei. **(B)** solid lamellar area of renal carcinoma: tumor cells arranged in solid lamellar shape, translucent cytoplasm, pyknotic/round nuclei, coarse chromatin, visible nucleoli. **(C)** sarcoma-like area of renal carcinoma: spindling cells, red-stained cytoplasm, coarse chromatin, visible nucleoli. **(D)** immunohistochemical PAX-8 nuclei, Positive expression.

On 23 February 2022, the patient underwent laparoscopic radical left kidney surgery. The pathology of our hospital showed the following: the tumor was consistent with renal cell carcinoma (**Figure 1**), with a histological grade of G3, but was not specifically classified; the tumor was located in the middle and lower pole of the kidney with a maximum diameter of about 8.5 cm, with local necrosis, sarcoma-like changes, tumor invasion of the perineurium, and no invasion of the renal sinus and perinephric fat; no tumor was seen at the ureteral end and renal tip end; and no lymph node was found. Immunohistochemistry: CAIX (−), CD10 (−), PAX-8 (+), TFE-3 (−), CAIX (−), CD117 (−), Vimentin (+), CK20 (−), CK34βE12 (−), CK7 (partial+), E-ca (+), INI1 (−), ki-67 index about 5%, P504s (+), and SDHB (+). Then, we had a consultation with the Cancer Center of Sun Yat-sen University and added KS-cad (−), Oct-4 (−), SALL-4 (−), and FH (+), which confirmed clear cell renal cell carcinoma grade 4, combined with sarcomatosis (10%) and necrosis (15%). Clear cell renal cell carcinoma with sarcomatoid, a clinically aggressive tumor, is characterized by rapid metastasis and a poor prognosis. The patient complained of pain in the right leg, then underwent a bone scan (PET-ECT) on 10 April 2022, which showed bone metastases in the left iliac bone and proximal right femur.

**Figure 2:**
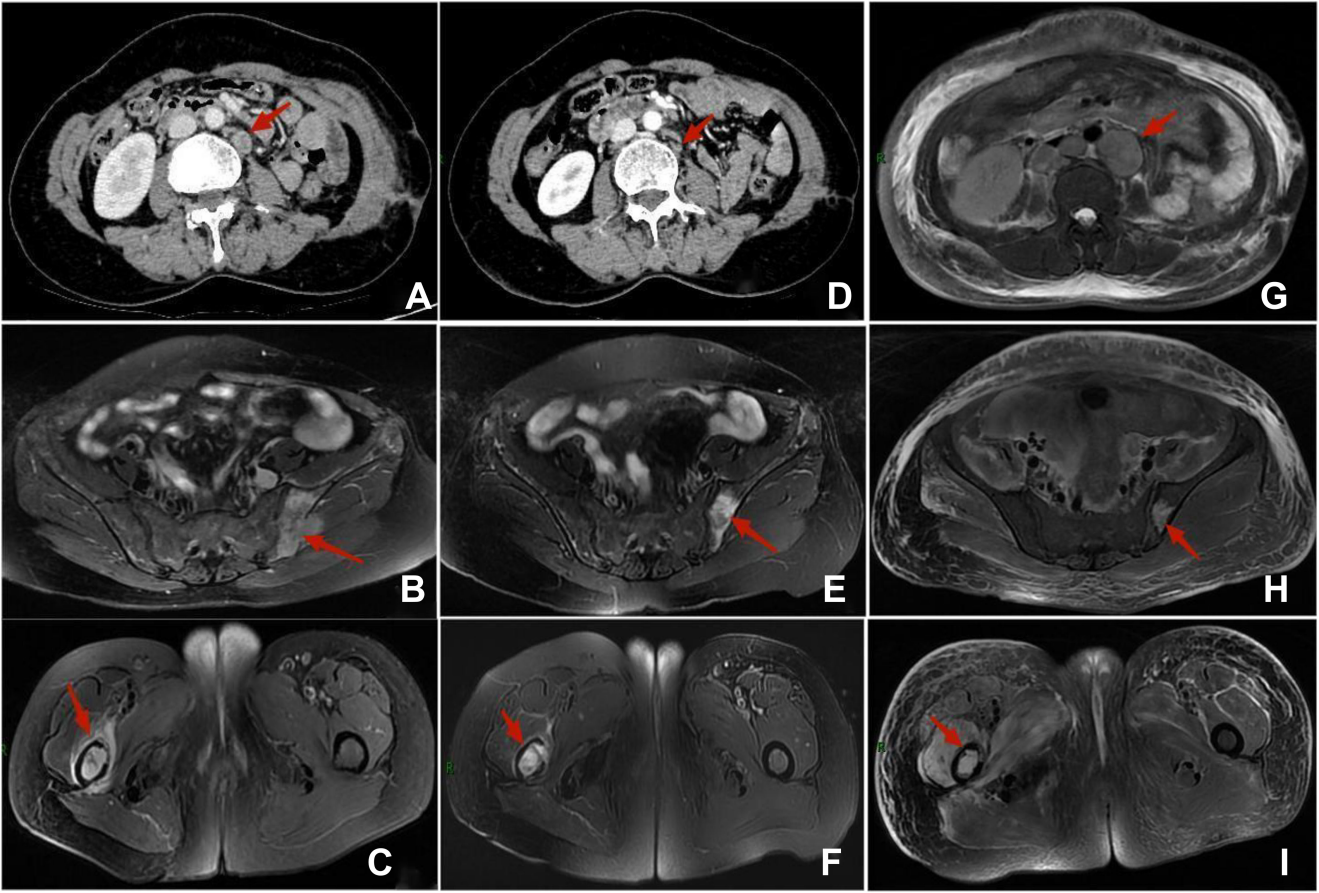
Before immune-targeted therapy: **(A)** Retrospective retroperitoneal parietal abdominal aorta and left iliac artery with several foci of slightly large round-like isointense nodules; the larger one is approximately 13 mm*11 mm. **(B)** Left iliac bone destruction with localized soft tissue shadow. **(C)** Right upper femur with local bone destruction and soft tissue shadowing. During immune-targeted therapy: **(D)** Reduced foci of round-like isodense nodules in the retroperitoneal abdominal aorta and left iliac artery; larger nodules are approximately 10mm*7mm. **(E)** Reduced soft tissue shadowing and less extensive bone destruction in the left iliac bone than before. **(F)** Similar foci in the right femur as before. Tumor progression: **(G)** Retroperitoneum with multiple enlarged lymph nodes adjacent to the abdominal aorta and fused with each other, the larger node being approximately 29mm*43mm. **(H)** The left iliac bone has a reduced soft tissue shadow, **(I)** the Lesion in the right femur is less extensive than before.

Our oncology department later consulted with the patient, and we carried out a thorough examination and pre-treatment imaging evaluation. On 14 April 2022, a color ultrasound scan of the thyroid gland showed a hypoechoic nodule measuring approximately 1.2 cm × 0.9 cm × 1.1 cm on the right lobe of the thyroid gland (2017 ACR score total: 11; TI-RADS category 5), which was considered to be thyroid cancer. Pelvic and abdominal CT revealed enlarged lymph nodes in the retroperitoneal abdominal aorta and left iliac artery (**Figure 2A**). A bone scan and MRI suggested bone metastases in the left iliac bone and proximal right femur (**Figures 2B, C**). The patient was diagnosed with clear cell renal cell carcinoma with sarcomatoid (pT2aN1M1, IV, AJCC 8th), and thyroid carcinoma through postoperative pathology and imaging.

**Figure 3:**
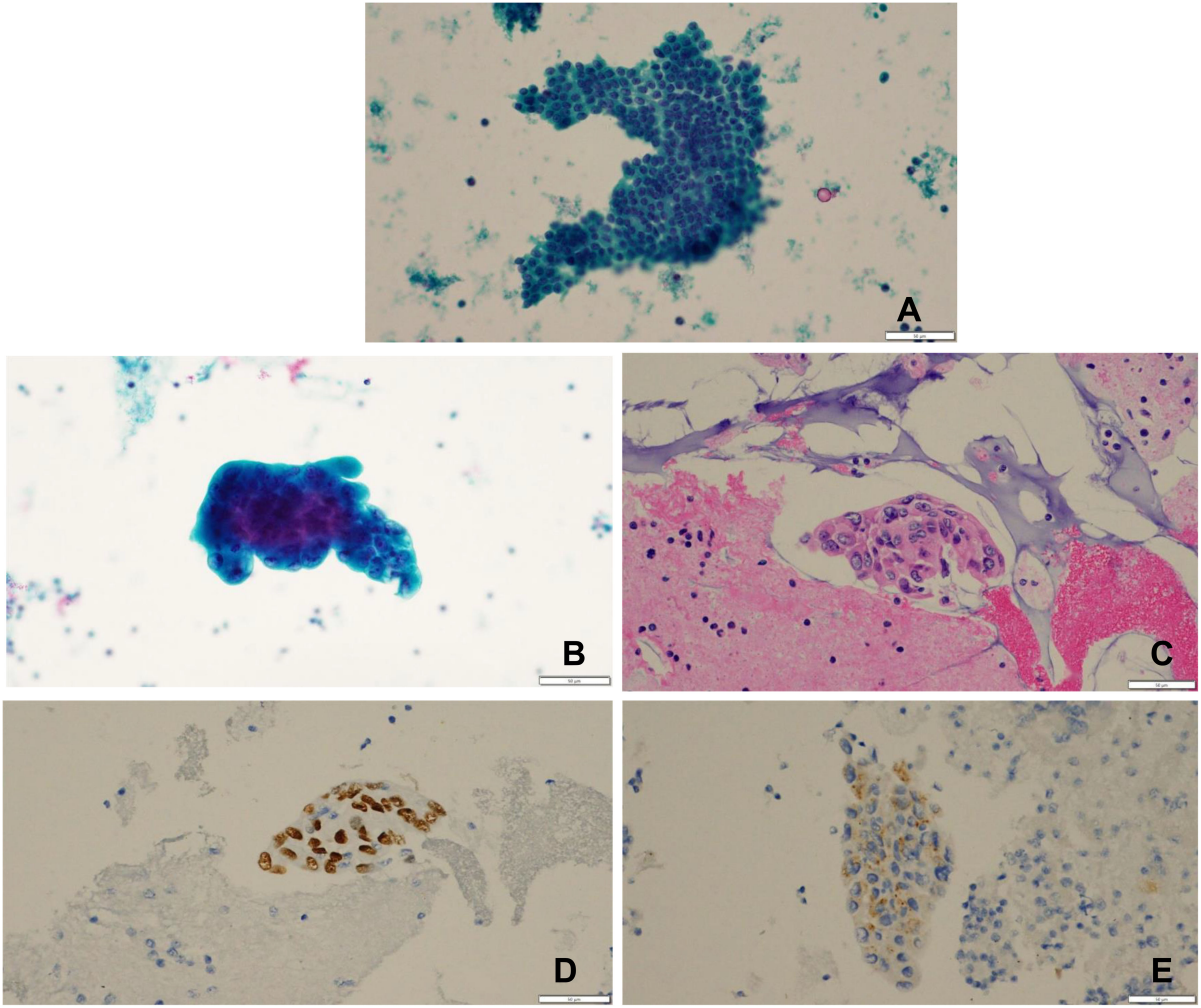
**(A)** under 400x microscope Thyroid cytology: Pap stained tumor cells were arranged in solid sheets with medium cytoplasm under 400x Pap staining, irregular nucleus morphology, vacuolated, chromatin border set, thickened nuclear membrane, and deviated nucleoli were seen, which are morphological features of typical papillary thyroid carcinoma. **(B)** Pap stain 400 times. **(C)** Picture shows one HE stain 400 times for cell wax block section.The tumor cells can be seen in clusters with three-dimensional distribution, rich cytoplasm, oval or irregular-shaped nuclei, vacuolated, with one or more large red nucleoli and visible nuclear division, which is consistent with the morphological characteristics of adenocarcinoma. **(D)** Immunohistochemistry TTF-1 positive expression, **(E)** Immunohistochemistry NapsinA positive expression positive expression of TTF-1 and NapsinA revealed a high probability that the tumor originated from the lung.

Thyroid ultrasound revealed hypoechoic thyroid nodules with calcifications in the upper pole and middle of the right lobe, as well as in the upper and lower levels of the left lobe; lymph nodes in the IV region of the left neck were irregular in shape and contained abundant blood flow signals. On 27 September 2022, the patient underwent fine needle aspiration of bilateral thyroid nodules and left neck IV lymph nodes. Thyroid cytology was consistent with the morphologic features of typical papillary thyroid carcinoma on the papillary stain (**Figure 3A**). The anisotropic cells of the neck lymph nodes matched cancer cells (**Figures 3B–E**). TTF-1 (+), PAX-8 (−), BRAF (−), P504s (−), TG (−), NapsinA (+), and vimentin (partial +) were the immunohistochemistry results of the neck lymph nodes. The chest CT suggested a ground glass nodule, and following treatment, the lesion shrank. We combined the pathological findings of lymph node cystocentesis of the neck, TTF-1, NapsinA positive expression, and probable pulmonary origin. The negative expression of PAX-8 and TG excluded a thyroid origin, and the negative expression of PAX-8 and P504s excluded a renal origin. We considered that the patient might have a third primary tumor present. Therefore, we advised the patient to have another neck lymph node aspiration biopsy and NGS testing to clarify the pathology and molecular typing and develop the next treatment strategy. Unfortunately, our patient refused this examination, leaving us without a reference for a definitive diagnosis of the third primary tumor.

### 2.3 Treatment

We obtained informed consent from patients and families for all of our treatments. The patient received one cycle of tirelizumab on 24 March 2022 outside the hospital. The patient then consulted our department and was evaluated for targeted combined immunotherapy. We did not provide targeted drugs considering that the patient might need to have a thyroid nodule puncture. On 15 April 2022, the patient received only immunotherapy. Later, in combination with the thyroid surgery consultation, we could monitor the change in the size of thyroid nodules. On 23 April 2022, the patient was started on oral lenvatinib.

Through multidisciplinary team discussion, the highest-grade malignancies are treated with priority; thus, our treatment focus at that time was on kidney cancer. Thyroid carcinoma could be considered for surgical treatment. However, the current thyroid cancer lesion did not significantly invade essential tissues and organs, such as the trachea and esophagus. We decided to treat clear cell renal cell carcinoma initially. Following the International Metastatic Kidney Cancer Database Consortium (IMDC) prognostic model for advanced renal cancer (5), three poor prognostic factors were identified in this patient, respectively: the interval between the diagnosis of primary renal cancer and systemic therapy was less than 1 year; hemoglobin was 85 g/L; and platelet count was 435 × 10^9^/L, so there was a high risk of stratification based on prognosis. According to NCCN guidelines, targeted combination immunization in advanced renal cancer (high-risk group) is the first-line recommended treatment option. Pembrolizumab combined with lenvatinib is the first-line agent (6). Considering the patient’s financial difficulties, we replaced pembrolizumab with the cheaper tirelizumab.

On 7 May 2022, the patient started treatment with an immune checkpoint inhibitor and a tyrosine kinase inhibitor (TKI), i.e., tirelizumab 200 mg iv drip Q3 week + lenvatinib mesylate 8 mg QD. The patient complained of pain in the right leg, and then a bone scan and MRI showed bone metastases in the left iliac bone and proximal right femur. From 11 May 2022 to 17 May 2022, the patient completed palliative radiotherapy using the stereotactic body radiotherapy (SBRT) technique with 8 MV photon lines at a prescribed dose of PGTV 30 Gy/5F. Subsequently, the patient had her first imaging evaluation after treatment in June 2022. According to the RECIST score, the tumor efficacy was classified as partial remission (**Figures 2D–F**). The patient’s right leg pain was relieved.

**Figure 4:**
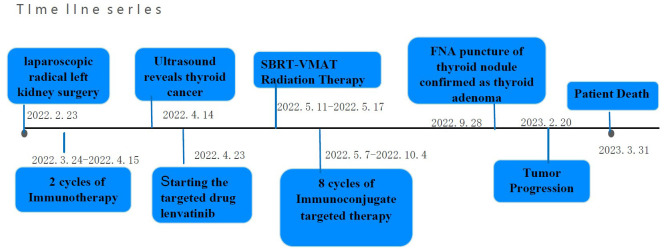
Timeline of the patient's entire treatment process.

On 20 September 2022, the patient underwent another post-treatment imaging and was stable according to the RECIST score. The patient had 10 months of progression-free disease. On 13 February 2023, the patient was admitted with bilateral lower extremity edema and was then found to have lower extremity thrombosis. Her joint MRI showed smaller left iliac and right femoral lesions than before, while abdominal MRI suggested new metastases in the liver and larger retroperitoneal lymph nodes than before, suggesting tumor progression (**Figures 2G–I**). We suggested a biopsy of the progressive lesion to assist with treatment, but the patient refused. At follow-up, the patient’s family informed us that she had died on 31 March 2023. The timeline of the patient's entire treatment process was depicted in **Figure 4**.

## 3 Discussion

MPMNs are rare. The reported incidence of MPMNs ranges from 0.7% to 11.7% (5). Second primary tumors in renal carcinoma with MPMN of other organs are most commonly detected in the genitourinary, digestive, and respiratory systems, such as bladder, lung, and colon cancers, while those combined with thyroid are less common. We report this patient to be of a different tissue origin from the kidney and thyroid, and in combination with immunohistochemical analysis, it meets the inclusion criteria for MPMN.

Although the exact mechanism of MPMN development is not yet known, the development of MPMN may be related to long-term smoking, exposure to UV exogenous factors, genetic susceptibility, endocrine and embryonic development, side effects of chemotherapy and radiotherapy, immunodeficiency, and age (6).

In one case, a patient was diagnosed with triple primary lung, kidney, and thyroid malignancies. EGFR and BRAF mutations were carried in lung and thyroid adenocarcinomas, respectively. This patient and her father, who was diagnosed with lung cancer, shared three potential germline cancer susceptibility mutations (7). However, the report did not mention specific treatment options for the three primary tumors, which differs from ours. Our patient had no family history of inheritance, and she did not undergo genetic testing because her family’s limited finances.

As medical treatment technology has improved, the incidence of MPMN has increased (2). Early diagnosis and multidisciplinary treatment may be the key factors affecting MPMN. Ishimori et al.’s (3) study has highlighted the importance of PET-CT imaging in identifying second primary tumors through the enhanced uptake of F18-FDG. Secondary lesions, not metastases or local spread, must be excluded in diagnosing a concomitant dual primary tumor. The combination of pathology and immunohistochemical analysis aids in differentiating metastatic or primary tumors. Once the diagnosis of a primary renal tumor has been confirmed, diagnostic imaging reveals suspicious thyroid lesions. It differentiates between primary and secondary thyroid cancer, using fine-needle aspiration (FNA) cytology with sensitivity and specificity to aid in differentiation (8). In this case, we found that the FNA cytology was consistent with the typical morphological features of papillary thyroid carcinoma.

Currently, there is no established treatment protocol for MPMN. The multidisciplinary team discusses individualized and comprehensive treatment based on tumor size, pathological type, and clinical stage. The highest-grade malignancies are treated with priority; surgery is the primary treatment option for patients who are able to undergo surgery; inoperable patients apply chemotherapy, targeted therapies, and other treatments to improve their quality of life (9). In conjunction with the patient’s condition, we performed a left nephrectomy based on the above principles and provided appropriate postoperative adjuvant therapy. Sarcomatoid renal cell carcinomas, which account for 1%–5% of all renal malignant neoplasms, are clinically aggressive tumors characterized by rapid metastasis and poor prognosis (10). Most trials report a poor median overall survival of 5 to 12 months (11). This patient had clear cell renal cell carcinoma with sarcomatoid, implying a poor prognosis. According to the predictive model analysis of the International Metastatic Kidney Cancer Database Consortium (IMDC), our patient belongs to the high-risk group (12). For advanced kidney cancer (high-risk group), targeted combination immunotherapy is the first-line recommended treatment option according to NCCN guideline recommendations (13). Based on the literature reported, we are the first to use tirelizumab combined with lenvatinib mesylate and palliative local radiation therapy for the management of synchronous kidney and thyroid cancer. However, the effectiveness of tirelizumab combined with lenvatinib in advanced kidney cancer still requires more clinical trials to validate.

In addition, lenvatinib is recommended for papillary thyroid cancer (14). Based on a phase II ATLEP trial (NCT02973997) published by the European Society of Medical Oncology (ESMO), the combination of lenvatinib with a PD-1 inhibitor improved the objective remission rate (ORR) and clinical benefit rate (CBR) (15). Our decision for this treatment option takes into account the treatment of both types of tumors.

One meta-analysis on the efficacy and safety of SBRT for metastatic renal cell carcinoma had an incidence of less than 1% for any grade 3–4 toxic reaction (16). The patient was treated with stereotactic radiotherapy as well as PD-1 inhibitors without an increase in overlapping toxicities. After SBRT combined with immunotherapy, bone metastases treated with radiation shrank, while iliac metastases not treated with radiation shrank, even after tumor progression, which is thought to be due to the distant effect of SBRT combined with immunotherapy. The patient later developed lower extremity thrombosis, new metastases in the liver, and larger than before retroperitoneal lymph nodes, suggesting tumor progression. We recommended a biopsy of the progressing lesions to assist in treatment. The patient was poorly compliant and refused the examination. Unfortunately, she finally died in March 2023. Despite this, the patient had a progression-free survival of 10 months.

In conclusion, we would like to express that the combination of tirelizumab with lenvatinib mesylate with SBRT showed good clinical efficacy in this female patient. However, this report is limited to a single-case experience with limitations. The efficacy of the treatment needs to be verified in more clinical trials.

## 4 Conclusion

Early diagnosis and individualized treatment implemented by a multidisciplinary team are crucial for patient prognosis, and the treatment of synchronous renal and thyroid double cancers using immunotherapy combined with targeted and local radiotherapy that we reported is effective and provides a valuable treatment strategy for similar patients in the future.

## Data availability statement

The original contributions presented in the study are included in the article/**Supplementary Material**. Further inquiries can be directed to the corresponding author.

## Ethics statement

Written informed consent was obtained from the individual(s) for the publication of any potentially identifiable images or data included in this article.

## Author contributions

YZ, ZX, MY, and SL designed the project. YT drafted the work. YZ, ZX, YT, XC, XL, and WL analysis of data for the work YZ, ZX, YT, XC, MY, XL, WL, and SL completed the revision work. All authors contributed to the article and approved the submitted version.
