# Supplementary material for: Synchronous multiple primary malignancies of clear cell renal cell carcinoma with sarcomatoid, thyroid carcinoma: a case report
Source: Front Oncol. 2023 Jun 27;13:1174306. doi: 10.3389/fonc.2023.1174306 (PMC10335400; doi:10.3389/fonc.2023.1174306)
Supplement: Supplementary file 1 [file Image_1.pdf]

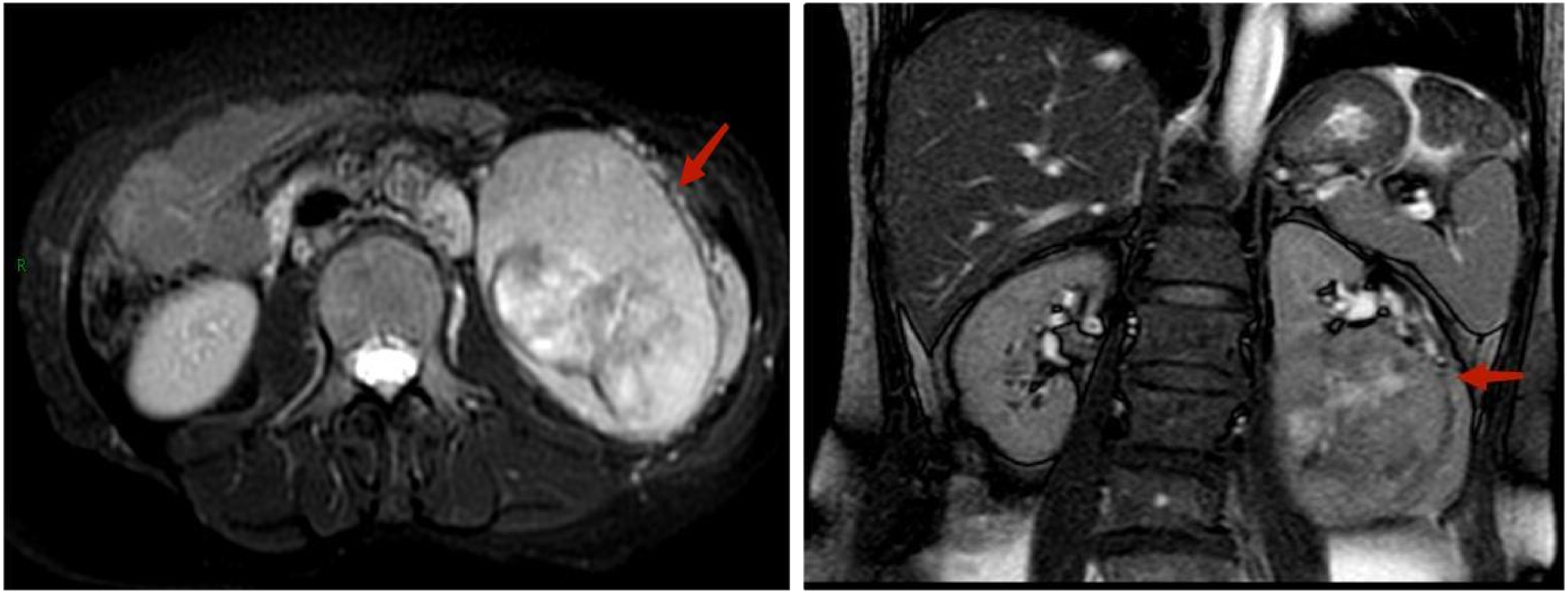


**Supplementary chart**

Preoperative abdominal magnetic resonance showed 70 mm × 96 mm × 81 mm round-like soft tissue mass in the lower and middle part of the left kidney, with the adjacent renal pelvis and calyces deformed by compression and a clear perirenal fatty space, and slightly enlarged retroperitoneal lymph nodes.
